# Supporting Tablet Configuration, Tracking, and Infection Control Practices in Digital Health Interventions: Study Protocol

**DOI:** 10.2196/resprot.5400

**Published:** 2016-06-27

**Authors:** Robert D Furberg, Alexa M Ortiz, Brittany A Zulkiewicz, Jordan P Hudson, Olivia M Taylor, Megan A Lewis

**Affiliations:** ^1^ RTI International Research Triangle Park, NC United States

**Keywords:** tablet computers, mHealth, infection control, clinical research protocol, mobile device management

## Abstract

**Background:**

Tablet-based health care interventions have the potential to encourage patient care in a timelier manner, allow physicians convenient access to patient records, and provide an improved method for patient education. However, along with the continued adoption of tablet technologies, there is a concomitant need to develop protocols focusing on the configuration, management, and maintenance of these devices within the health care setting to support the conduct of clinical research.

**Objective:**

Develop three protocols to support tablet configuration, tablet management, and tablet maintenance.

**Methods:**

The Configurator software, Tile technology, and current infection control recommendations were employed to develop three distinct protocols for tablet-based digital health interventions. Configurator is a mobile device management software specifically for iPhone operating system (iOS) devices. The capabilities and current applications of Configurator were reviewed and used to develop the protocol to support device configuration. Tile is a tracking tag associated with a free mobile app available for iOS and Android devices. The features associated with Tile were evaluated and used to develop the Tile protocol to support tablet management. Furthermore, current recommendations on preventing health care–related infections were reviewed to develop the infection control protocol to support tablet maintenance.

**Results:**

This article provides three protocols: the Configurator protocol, the Tile protocol, and the infection control protocol.

**Conclusions:**

These protocols can help to ensure consistent implementation of tablet-based interventions, enhance fidelity when employing tablets for research purposes, and serve as a guide for tablet deployments within clinical settings.

## Introduction

The proliferation of tablet technologies has ushered in a new realm of connected health care and research-based interventions in the clinical setting. According to the Pew Research Center, 64% of American adults own a smartphone, and of those, 53% also own a tablet [1]. Advancements in policy, health information technology adoption by providers, mobile/social technology adoption by consumers, and development of health-focused mobile apps have created ideal conditions to enable the expansion of tablet-based interventions. According to the *2015 Healthcare Information and Management Systems Society Mobile Technology Survey*, 47% of responding organizations labeled mobile service implementation to access information as a top priority, and 57% indicated already having a mobile technology policy [2]. Further, as noted by the IMS Institute for Healthcare Informatics, more than 165,000 mobile health (mHealth) apps are currently available for consumer download through Google Play and the Mac App Store [3].

The use of electronic tools and services has created new opportunities for individuals to actively participate in monitoring and directing their health care through digital health interventions. The body of evidence that supports the use of such strategies to improve health outcomes continues to expand [4]. However, insufficient guidance exists on deployment methods that could be used to support replication or subsequent scaling. To address this gap, we describe protocols relevant to three major phases of digital health intervention activities using tablets: initial device configuration, remote management of deployed technologies, and the ongoing maintenance of tablets deployed within clinical environments. The protocols are intended for use by three major audiences: (1) public health investigators responsible for the design of a digital health intervention, (2) research technologists seeking guidance on deployment methods, and (3) clinical staff responsible for monitoring tablets and carrying out infection control procedures in a health care setting.

There is scant literature describing the implementation of tablet-based public health interventions and the management of tablets. Tablet use takes place across multiple populations and settings including urban ambulatory care practices, grocery stores, pediatric practices, and primary care practices [5-8]. Several interventions describe the use of Web-based material optimized for delivery via tablets, whereas others used operating system–specific tablet-based interventions [7-13]. Irrespective of the mode of content delivery (eg, native app, hybrid app, or responsive Web application), proper configuration, management, and maintenance of the tablet computer is essential to ensure technical fidelity and control of participant exposure to intervention materials. To the best of our knowledge, no studies describe these procedures, and there are no predeveloped protocols surrounding the use of tablets in digital health interventions. We describe protocols to address the gap within the literature that can serve as a basic guide for configuration, management, and maintenance of tablet-based interventions.

## Methods

### Tablet Configuration

#### Characteristics of Configurator

The mobile device management software, known as Configurator, is a resource that may improve the reliability and enhance the reproducibility of digital health interventions. Configurator is a free utility available from the Mac App Store. It is designed to configure and deploy multiple iPhones, iPod Touches, iPads, or Apple TV devices [14]. Configurator users have the ability to push images, settings, apps, and security profiles across groups of iPhone operating system (iOS) devices [15]. These devices can be configured using an XML configuration file or by using the backup of a master iPad [16,17], which is configured by hand. The configuration can include all network and security settings as well as installs for the selected free or in-house apps. Once set, the master iPad is imaged using Configurator’s backup function [16]. Remaining iPads are connected to the Mac OS device via universal serial bus (USB), and the image of the master iPad is restored to the remaining iPads. This can be done with up to 30 iPads [15]. Using Configurator to set up and maintain iOS devices gives administrative control over every aspect of the device, including wallpaper and lock screen settings, virtual private network and Wi-Fi (wireless local area network) settings, mail and calendar accounts, iOS version and upgrades, documents, and application access [16]. The end result is a number of identical, incrementally identified iOS devices. Subsequent alterations, including software updates, can only be made using Configurator by connecting the devices via USB to a Mac OS computer.

#### Applying Configurator to Tablet Deployment

Presently there is limited guidance available regarding tablet configuration for digital health interventions. However, with reported sales of more than 4.5 million iPads to educational institutions within the United States and almost twice that worldwide, Configurator is extensively used within the field of education [18,19]. Recommendations from Configurator’s application in education can be applied to digital health interventions deployed for research or clinical purposes.

The Configurator application is widely used in education for configuring multiple iOS-based devices at scale, whether it be for a single classroom, school, or district. Consequently, a considerable amount of guidance is available about Configurator’s setup and use in schools. Additionally, the Configurator education deployment model provides institutions with flexibility to manage multiple deployment scenarios, such as one-to-one, shared-use, and student-owned devices. Schools may choose to deploy iPads in one specific scenario or all three scenarios. However, Configurator is ideally used to set up one-to-one or shared-use devices, which gives educational IT staff the ability to customize and be flexible in addressing the school’s specific needs.

**Figure 1:**
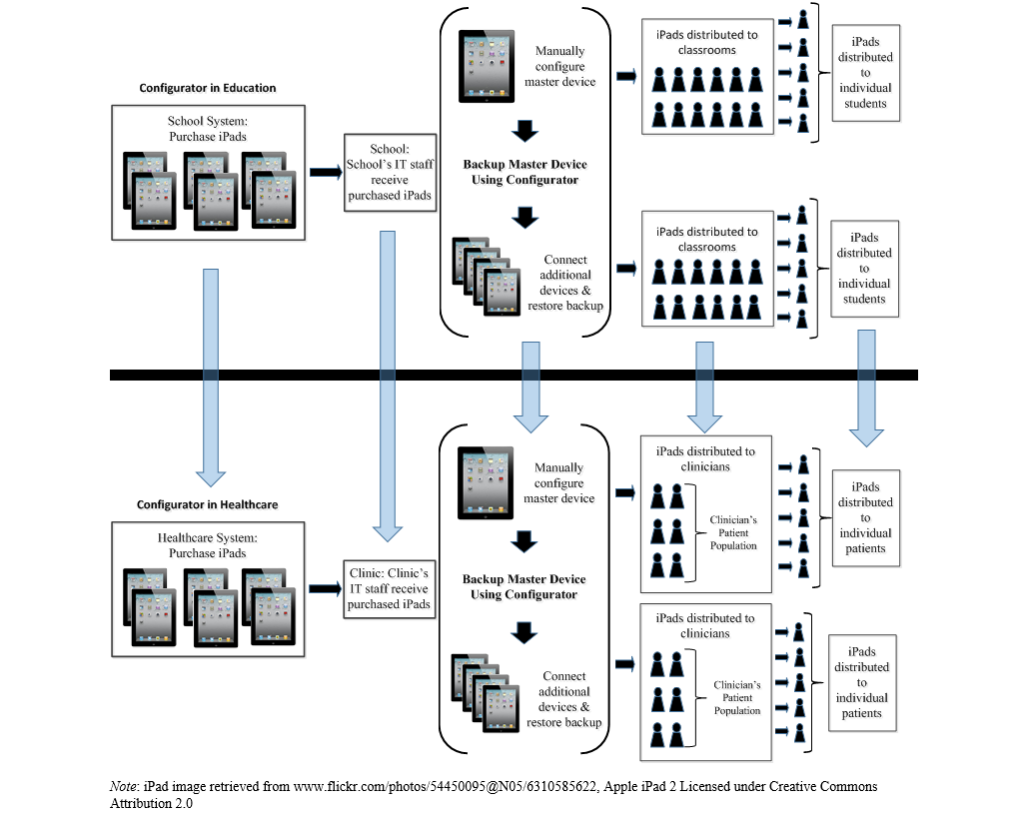
Education and healthcare comparative configurator infrastructure.

The iOS education Configurator deployment model can easily be transposed to other domains, such as deployment within clinical settings or health research studies. The example provided in Figure 1 outlines how the educational infrastructure is similar to the health care infrastructure, as can be seen by changing the nomenclature from schools to clinics, teachers to clinicians, and students to patients.

#### Configurator Protocol

As the use of tablets continues to expand within clinical settings and tablets become more commonplace components in digital health interventions, a consistent method for configuring devices will become increasingly necessary. The protocol, as shown in Multimedia Appendix 1, provides a generic outline for iPad deployment using Configurator within research or clinical settings. The protocol reviews the requirements associated with Configurator’s use and provides the steps for setting up an iPad using Configurator.

### Tablet Management

#### Characteristics of Tile

**Figure 2:**
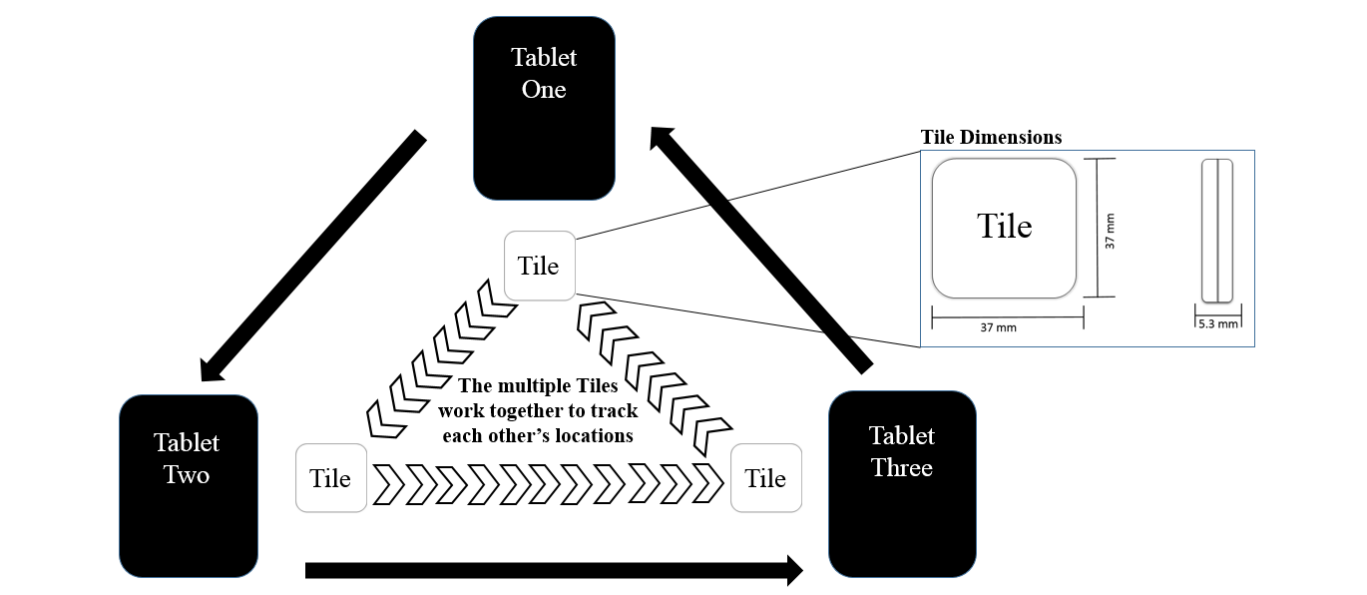
Entity relationship diagram: option 1 and tile dimensions.

The ability to track tablets (or other mobile devices) is an investment in the retention of the digital health intervention. Tile is a small tracking tag (see Figure 2 for dimensions) associated with a free app compatible with iOS and the Android operating system [20-23]. The primary purpose of Tile is to assist users in locating lost items. While Tile was not designed for mobile device management, its capabilities and small size offer a unique method of tracking mobile devices in clinical settings to augment native management features, such as Find My iPhone, where a Global Positioning System (GPS) signal may not be available.

The Tile app is free and the cost of Tile’s hardware is relatively low—usually less than $25 per device [24]. Tile locates items by using Bluetooth low energy (BLE) 4.0 [22,23]. BLE works optimally at a range of 30 feet but can extend to a maximum range of 100 feet [22,23]. If an item is lost but is in the range of the BLE, Tile can be triggered to play a short tune by selecting the *Find* option within the app to assist the user in locating the lost device [23,25]. If Tile is unable to track an item via BLE, the user can select the app’s *Mark as Lost* option to report a missing Tile [23]. The app can also inform users about the last place their lost device was seen. As noted in the Tile informational material [23], “Tile maps the last place you had it.”

Additionally, multiple Tiles can work together. While an individual Tile is only visible to its registered user, each Tile can be used in a *Community Find*, which allows any Tile to assist in communicating the location of a lost item to its registered owner [22,25,26]. Separate from the *Community Find* feature, the Tile app can share a Tile’s location with another Tile app user [27]. The *Share* feature allows two different app users access to the same Tile [23,28].

#### Applying Tile to Tablet Deployment

There are two approaches to applying Tile technology to tablet deployments. The first option, as outlined in Figure 2, displays three devices that each track one other device within the diagram. Since each Tile’s BLE can only connect to one device at a time [27], each tablet is limited to tracking only one other tablet. That is, tablet 1 is used to track the location of tile 2 (attached to tablet 2); tablet 2 is used to track the location of tile 3 (attached to tablet 3); tablet 3 is used to track the location of tile 1 (attached to tablet 1).

**Figure 3:**
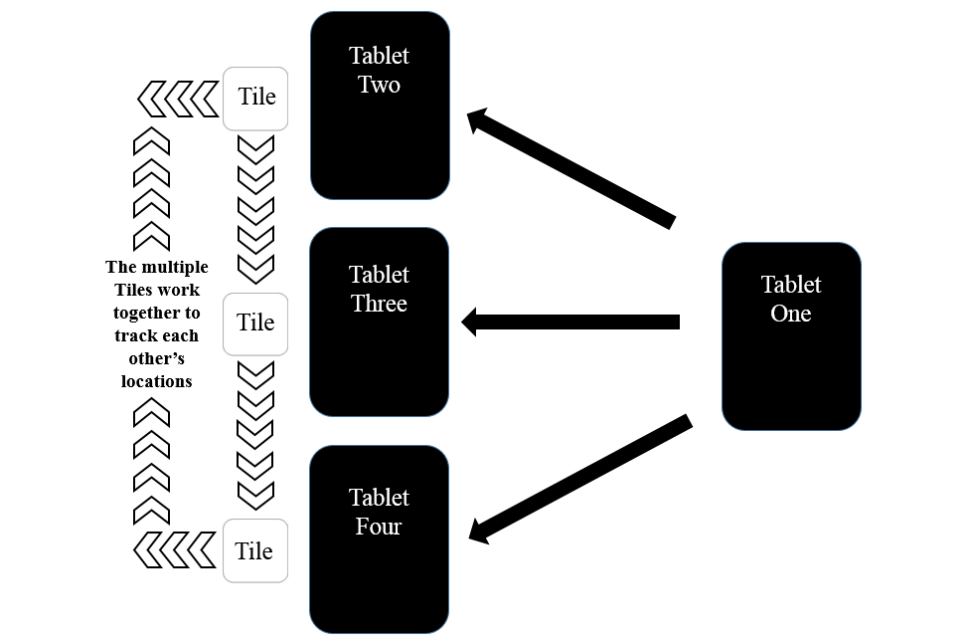
Entity relationship diagram: option 2.

The second option for applying Tile technology to tablet deployments is outlined in Figure 3, which displays four devices, three with Tiles attached and one device that is solely used for tracking the deployed Tiles. Because one Tile app can simultaneously track multiple individual Tiles, a single tablet can track the three deployed Tiles via BLE; that is, tablet 1 can track tablets 2, 3, and 4.

#### Tile Protocol

Considering the compact nature of many tablets, the risk of theft, misplaced devices, or loss is a concern. Consequently, enhanced use of tablet technology in digital health interventions must be accompanied by innovative methods of device management. Multimedia Appendix 2 provides a protocol outlining the requirements for deployment as well as how to set up and use the various features offered by the Tile tracking device.

### Tablet Maintenance

#### Characteristics of Infection Control

As tablets continue to proliferate the health care setting, the risk of device-based pathogen transmission increases [29]. In a 2009 study, the mobile phones of 200 intensive care and operating room health care workers were tested, and 94.5% showed some form of bacterial contamination [30]. Although the study does not discuss tablets, the same concerns of bacterial contamination and infection surrounding mobile phones are applicable to tablets.

Presently, Apple recommends using a soft, lint-free cloth for cleaning mobile devices, avoiding liquids, solvents, or spraying cleaners directly onto the device [31]. However, a plain cloth will not remove contagious pathogens such as methicillin-resistant *Staphylococcus aureus* (MRSA) or vancomycin-resistant enterococcus (VRE) from a mobile device [29]. A study by Howell and colleagues [29] compared six different wipes (Sani-Cloth CHG 2%, Trigene, Clorox, Tristel, soap and water, and a plain cloth) and found that the Sani-Cloth CHG (chlorhexidine gluconate 2%/alcohol 70%) wipes were the most effective cleaning agent for tablets, with very low risk to the tablet’s functional capabilities or accessories.

#### Infection Control Protocol

Proper disinfection is an important component to consider as tablets are increasingly integrated into both research studies and patient care. The infection control protocol is reviewed in Multimedia Appendix 3 and provides a list of the required supplies as well as an outline of the steps needed to disinfect tablet devices using CHG 2% wipes.

## Results

This article provides three protocols within the multimedia appendices that can be applied to support the configuration, the management, and the maintenance of mobile devices employed in digital health interventions: the Configurator protocol, the Tile protocol, and the infection control protocol.

## Discussion

This article addresses the literature gap on tablet protocols used in digital health interventions and describes three protocols supporting tablet configuration, management, and maintenance. The protocols discussed are furnished as generalized instructions or as a foundation for further enhancements, allowing for customization to further optimize tablet adoption. While these protocols can be used to support a single intervention, the resources may be used individually or bundled, at the discretion of investigators or clinicians, depending on the methodological requirements and deployment environment.

The availability and utilization of tablets is advancing the way researchers work, clinicians provide health care, and patients receive health care. However, along with the proliferation of tablets comes the need to advance tablet-focused protocols. The goal of this paper was not to test the effectiveness of Configurator, Tile, or the proposed infection protocol. Rather, we sought to review various options available to augment consistent tablet configuration, management, and maintenance. Further research is necessary to evaluate the feasibility and efficacy of the developed tablet-focused protocols.

## Appendix

Multimedia Appendix 1 Configurator Protocol.

Multimedia Appendix 2 Tile Protocol.

Multimedia Appendix 3 Infection Control Protocol.

## Abbreviations

BLE: Bluetooth low energy
CDC: Centers for Disease Control and Prevention
CHG: chlorhexidine gluconate
GPS: Global Positioning System
iOS: iPhone operating system
mHealth: mobile health
MRSA: methicillin-resistant *Staphylococcus aureus*
OS: operating system
PPE: personal protective equipment
USB: universal serial bus
VRE: vancomycin-resistant enterococcus
Wi-Fi: wireless local area network technology
