# Supplementary material for: Supporting Tablet Configuration, Tracking, and Infection Control Practices in Digital Health Interventions: Study Protocol
Source: JMIR Res Protoc. 2016 Jun 27;5(2):e136. doi: 10.2196/resprot.5400 (PMC4940603; doi:10.2196/resprot.5400)
Supplement: Multimedia Appendix 1 [file resprot_v5i2e136_app1.pdf]

Multimedia Appendix 1: Configurator Protocol

Requirements

- Tablet with iOS 8 or later
- A computer with:
  - OS X v10.10.5 or later
  - iTunes 12.3.1.23 or later
  - Apple Configurator 1.7 or later

Setting Up an iPad Using Apple Configurator

1. Make sure there is no /Users/Shared/SC Info folder.
2. Create Apple ID with no credit card, based on generic email.
3. Authorize iTunes Store with new Apple ID.
4. Make sure that Apple ID is signed into store.
5. Open Apple Configurator.
6. Under Prepare, select Settings and set “Supervision” to “On.”
7. Select “Prepare.”
8. Once supervision is complete, unplug the iPad and complete the iOS setup process on that iPad.
9. Select English.
10. Select United States.
11. Select Enable Location Services.
12. Connect to wireless network.
13. Setup as new iPad.
14. Skip Apple ID steps by selecting “Skip This Step.”
15. Accept Terms and Conditions.
16. Choose whether to automatically send Diagnostics & Usage Data.

17. Plug back into Apple Configurator.

18. Under Supervised section select iPad.

19. Under Settings, go to the “Restore” drop down box.

20. Select “Back up.”

21. Name the back up.

22. Once complete, unplug the device.

23. Now, plug in the other iPads, one at a time so the names get applied sequentially according to position in order.

24. Under Prepare select Settings and set “Supervision” to “On.”

25. Under the Restore drop down box select the backup created in step 21 above.

26. Select prepare.

27. Finally, move to the Supervise pane and select the profiles and applications to install on devices.

28. Select “refresh” to complete configuration.
