# Supplementary material for: Supporting Tablet Configuration, Tracking, and Infection Control Practices in Digital Health Interventions: Study Protocol
Source: JMIR Res Protoc. 2016 Jun 27;5(2):e136. doi: 10.2196/resprot.5400 (PMC4940603; doi:10.2196/resprot.5400)
Supplement: Multimedia Appendix 2 [file resprot_v5i2e136_app2.pdf]

Multimedia Appendix 2: Tile Protocol

Requirements

- Tablet(s) running the Tile App
- Tile Tracking Devices

Setting Up a Tablet with Tile

1. Download the free Tile app from either Apple or Google Play.

2. Open the Tile app.

3. Select “+” within the Tile app to add the Tile to a specific tablet. This tablet will now be the owner of that Tile.

4. Press the “e” button on the Tile device. This will trigger the Tile to play a tune.

5. Once the Tile’s tune has been heard, the Tile can be placed directly atop the tablet (a picture will appear on the tablet’s screen to guide the user).

6. At this point, the Tile can be named based on the item/tablet it may be tracking. In addition, a picture can also be uploaded to the Tile app of the item/tablet a specific Tile is tracking.

Making the Tile Ring

1. Open the Tile app and select the Tile that you would like to make ring.

2. In order to ring to a Tile, it must be within range of the BLE. When within range, a green circle will appear around the selected Tile’s icon within the Tile app. If a Tile is not within range of the BLE, please move to marking an item as lost.

3. If within range of the BLE, Select “Find”: The Tile should begin to ring.

4. Once the missing item/tablet has been located, select “Done” within the Tile app to stop the Tile from continuing to ring.

Marking an Item as Lost

1. Open the Tile app on the tablet that owns the lost Tile.

2. Select the tracking Tile that is attached to the lost item/tablet.

3. Select “Mark as Lost.”

4. When the lost Tile is located, a push notification stating the lost Tile was found will appear on the owner’s tablet.

5. The Tile app can then be opened to review the location of the lost Tile.
