# Supplementary material for: Supporting Tablet Configuration, Tracking, and Infection Control Practices in Digital Health Interventions: Study Protocol
Source: JMIR Res Protoc. 2016 Jun 27;5(2):e136. doi: 10.2196/resprot.5400 (PMC4940603; doi:10.2196/resprot.5400)
Supplement: Multimedia Appendix 3 [file resprot_v5i2e136_app3.pdf]

Multimedia Appendix 3: Infection Control Protocol

Requirements

- Tablet (may have cover)
- PPE: Gloves
- CHG 2% wipes
- Hand Washing Supplies: Water, Soap, Drying Agent

Disinfecting a Tablet

Warning: Contact poison control if any of the CHG 2% solution gets into eyes or is swallowed.

1. Prior to initiating the tablet cleaning protocol, the individual should thoroughly wash and dry their hands.

2. After the user's hands have been cleaned they should then power down the tablet and unplug all attached cables from the device.

3. If applicable, remove cover from tablet.

4. Apply Personal Protective Equipment – Gloves.

5. Take Sani-Cloth CHG 2% wipe and remove any extra liquid. The wipe being utilized to clean the tablet should not be dripping; however, it should be damp enough to clean the entire device.

6. Wipe down both the front and back of the device.

7. After the entire device is wiped down with the CHG 2% solution, it should be left to sit for 5 minutes until completely dry.

8. If applicable, take another Sani-Cloth CHG 2% wipe and clean the tablet cover (both front and back).

9. If applicable, allow the cover to sit for 5 minutes until completely dry.

10. If applicable, once both the tablet and cover are dry, the two can be placed back together and the device may now be powered back on.

11. This process should be completed on a daily basis and as needed when the tablet or cover is visibly soiled.

12. After the device has been cleaned, the individual cleaning the device should thoroughly wash and dry their hands.
